# Peroxodisulphuric acid synthesis in a flow electrolyser and its potential utilisation for black mass leaching

**DOI:** 10.1039/d5ra06474k

**Published:** 2025-11-03

**Authors:** Aigerim Tazhibayeva, Altynai Tanash, Yaroslav Zhigalenok, Saken Abdimomyn, Seiilbek Malik, Kaiyrgali Zhumadil, Sergey Nechipurenko, Fyodor Malchik

**Affiliations:** a Al-Farabi Kazakh National University, Department of Chemistry and Chemical Technology Almaty Kazakhstan frodo-007@mail.ru

## Abstract

This study demonstrates the electrochemical synthesis of peroxodisulfuric acid (H_2_S_2_O_8_) in a coaxial flow-type electrolyser. It evaluates its potential as a leaching agent for the black mass from spent lithium-ion batteries. The optimised synthesis (conditions: flow rate, current density) achieved high concentrations of (H_2_S_2_O_8_) (≈180 g dm^−3^) at a specific energy consumption of nearly 1.5 Wh g^−1^. The leaching performance of H_2_S_2_O_8_ was compared with that of conventional systems, including aqua regia and 2 M H_2_SO_4_ + H_2_O_2_. While aqua regia completely dissolved the NMC phase, and the H_2_SO_4_/H_2_O_2_ mixture ensured nearly full transition metal leaching, H_2_S_2_O_8_ leaching resulted in only partial dissolution of Ni (≈61%), Co (≈61%), and Mn (≈5%). However, lithium was fully extracted (≈99.6%) due to dual dissolution from residual electrolyte salts and chemical deintercalation from the cathode lattice. Mechanistic analysis using XRD, AAS, and Pourbaix diagrams revealed that the poor transition metal recovery originates from the extreme oxidising environment of H_2_S_2_O_8_, which stabilises insoluble high-valent oxides and prevents reductive dissolution pathways. The results highlight that direct application of H_2_S_2_O_8_ is less practical than H_2_SO_4_/H_2_O_2_ for transition metal extraction but could be exploited for selective Li recovery or integrated into a three-step process: (i) *in situ* H_2_SO_4_/H_2_O_2_ generation, (ii) controlled hydrolysis to H_2_SO_4_ + H_2_O_2_, and (iii) reductive leaching. This approach offers industrial advantages, including on-site oxidant production and the elimination of H_2_O_2_ transport hazards.

## Abstract 2

A coaxial flow electrolyser generates peroxodisulfuric acid at 180 g L^−1^ concentration with 1.5 Wh g^−1^ energy consumption. Applied to spent battery black mass, the acid extracts lithium quantitatively but shows limited transition metal recovery.

## Graphical abstract

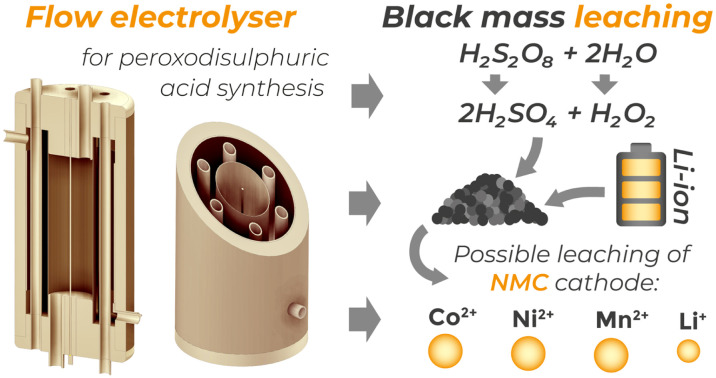

## 1 Introduction

Peroxodisulfuric acid (H_2_S_2_O_8_), also known as Marshall's acid, is a strong oxidizing agent (*E*° = 2.01 V *vs.* NHE) belonging to the family of sulfur peroxoacids. It contains an O–O peroxo bridge linking two –SO_3_H groups. The industrial production of H_2_S_2_O_8_ dates back to the early 20th century, with the first plant established in Weissenstein, Austria, in 1908. Three main methods are known: (i) the Levenstein process (electrolysis of ammonium sulfate in sulfuric acid), (ii) the electrolysis of sulfuric acid or sulfate solutions using platinum anodes, and (iii) improved processes employing advanced anode materials to suppress oxygen evolution.^1–3^

According to most researchers, the oxidation of sulfuric acid and its salts on electrodes made of platinum and platinum group metals proceeds *via* an electrochemical mechanism and, according to current data, consists of several main stages:^4^1
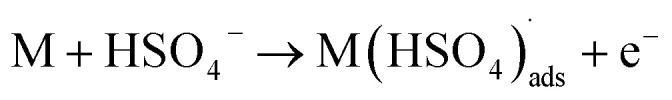
2
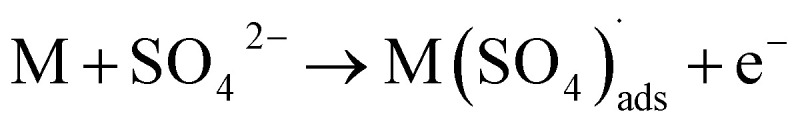
3
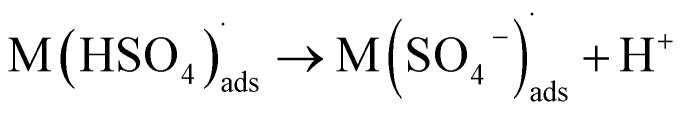
4
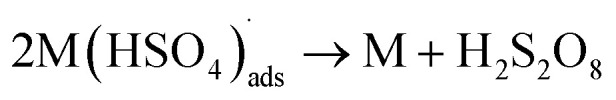
5
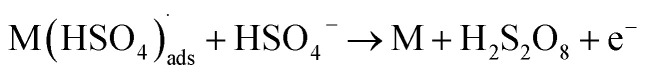


The first stage is the adsorption (on metal surface M) and discharge of sulfate or bisulfate ions *via* reaction (1) or (2). Since, in the electric double layer field, the adsorbed bisulfate radical can undergo deprotonation to form chemisorbed sulfate anion radicals *via* reaction (3), it is fundamentally unimportant whether sulfate or bisulfate ions are discharged, as both are present in the solution; an equilibrium state will be established. The removal of these intermediate species occurs through subsequent recombination reactions (4) or electrochemical desorption (5).^4^ H_2_S_2_O_8_ and its salts (Na_2_S_2_O_8_, K_2_S_2_O_8_, (NH_4_)_2_S_2_O_8_) are widely used in industrial processes such as polymerisation initiation, etching of printed circuit boards, oxidative wastewater treatment, textile bleaching, and chemical oxidation of recalcitrant organic pollutants.^4–7^

In recent years, persulfates have also found application in hydrometallurgy, including the oxidative leaching of valuable metals from black mass from spent lithium-ion batteries (LIBs). Recycling of end-of-life batteries is essential for both environmental protection and resource conservation. Spent batteries contain valuable metals such as lithium, cobalt, nickel, and manganese, whose extraction from primary ores is energy-intensive and environmentally damaging.^8^ At the same time, improper disposal of batteries can lead to the release of toxic electrolytes and heavy metals into soil and water, posing risks to ecosystems and human health.^9^

For example, Ji *et al.* proposed a lithium-selective extraction from spinel-type LiMn_2_O_4_ based on the hydrolysis of Na_2_S_2_O_8_.^10^ At 35 °C, the extraction ratio of lithium is no more than 10%, and the loss of manganese is about 2%. At 75 °C, the extraction ratio of lithium rapidly reaches over 93% almost without manganese loss, which is attributed to the inhibiting effect of 
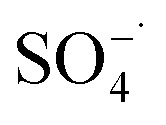
 free radicals. A similar scenario with lithium extraction of nearly 96% was achieved using different persulphate salts (NH_4_^+^, K^+^, Na^+^) at a temperature of 80 °C with Mn dissolution rate of 24–26%.^11^ The Na_2_S_2_O_8_, or as the authors name the process, advanced oxidation coupling with chemical leaching, was successfully used to achieve the selective extraction of Li from spent NCM523 scraps.^6^ About 91.23% Li was extracted from this black mass, with only 17.56% Ni leached using mild optimised conditions.

Nevertheless, the presented studies were primarily aimed at selective lithium recovery. At the same time, other valuable metals, especially in multicomponent cathode materials, remain in the solid phase and require additional pyro- or hydrometallurgical steps for their separation. The direct application of H_2_S_2_O_8_ is still debatable due to its high standard oxidation potential (*E*° = 2.01 V *vs.* NHE), which is sufficient to oxidize couples such as Co^2+^/Co^3+^, Ni^2+^/Ni^3+^, or Mn^2+^/Mn^4+^, but does not facilitate the reduction of high-valent, insoluble metal forms to their lower-valent, soluble states. However, H_2_S_2_O_8_ undergoes hydrolysis with the formation of H_2_O_2_ (6, 7) and H_2_SO_4_, which is a well-known reductive couple for leaching metal from black masses.^12,13^

6 H_2_S_2_O_8_ + H_2_O → H_2_SO_5_ + H_2_SO_4_

7 H_2_SO_5_ + H_2_O → H_2_O_2_ + H_2_SO_4_

The hydrolysis process is relatively slow, and a portion of the generated H_2_O_2_ can undergo immediate catalytic decomposition (8). Nevertheless, H_2_S_2_O_8_ can potentially be adopted for metal leaching from black mass, particularly when the acid is generated on-site (*e.g.*, at a recycling facility) *via* a simple electrochemical process, considering both achievable product concentration and cost efficiency. From a mechanistic perspective, *in situ* electrochemical generation of H_2_S_2_O_8_ in a flow electrolyser offers key advantages, including minimisation of storage and transport losses of potentially dangerous H_2_O_2_ (in concentrated state).

This article aims to present an effective method for H_2_S_2_O_8_ generation using an innovative coaxial-configuration flow electrolyser to optimise the synthesis conditions for reduced energy consumption and to demonstrate the potential application of peroxodisulfuric acid as a leaching agent for the extraction of valuable metals from real black mass supplied by an operating LIB recycling facility.

## 2 Experimental

### 2.1 Materials

The following reagents were used in this work: H_2_SO_4_, *ρ* = 1.8 g cm^−3^, pure grade; H_3_PO_4_, concentrated, analytical grade; NH_4_CNS, analytical grade; Fe(NH_4_)_2_(SO_4_)_2_·6H_2_O, analytical grade, Fixanal 0.1 N; KMnO_4_, Fixanal 0.1 N; H_2_O_2_, 37%, analytical grade; HNO_3_, analytical grade; HCl, 37% analytical grade; black mass (NMC + graphite) was donated by a local battery recycling company LLP « First Recycling ».

### 2.2 Equipment

The crystal structures of the samples were analysed by X-ray diffraction (XRD, Tongda TD-3700, China) with Cu Kα radiation (*λ*_1_ = 1.54056 Å, *λ*_2_ = 1.54439 Å). XRD patterns were collected in the 2*θ* range of 10–80. Metal concentration in the leaching solution was determined by an atomic absorption spectrometer AA-6200 (Shimadzu, Japan).

### 2.3 Experimental methods

Method for determining the concentration of peroxodisulfuric acid. The peroxodisulfuric acid content in the sample was determined by titration using a back-titration method. In this approach, the analyte (H_2_S_2_O_8_) reacts with an excess of Fe^2+^ (reducing agent), and the remaining Fe^2+^ is titrated with potassium permanganate. First, a blank sample containing Fe^2+^ (Mohr's salt, Fe(NH_4_)_2_(SO_4_)_2_·6H_2_O)) of known concentration was titrated with KMnO_4_. Specifically, 10 cm^3^ of 0.1 mol dm^−3^ Mohr's salt solution was transferred into a flask, followed by the addition of 5 cm^3^ of 20 wt% H_2_SO_4_ and 2 cm^3^ of concentrated H_3_PO_4_ to complex the trace amounts of Fe^3+^ present in Mohr's salt. The solution was titrated with 0.1 N KMnO_4_ until a stable pink endpoint was reached. Next, the working sample containing 1 cm^3^ of the analyte and 10 cm^3^ of Fe^2+^ solution was titrated under the same conditions.

The mass of H_2_S_2_O_8_ was calculated using the equation:8
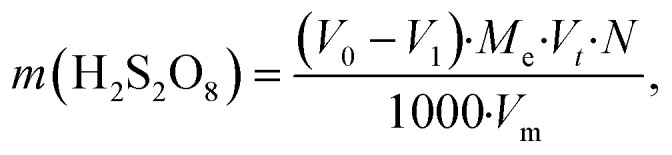
where *m*(H_2_S_2_O_8_) is the mass of peroxodisulfuric acid produced during electrolysis, g; *V*_0_ is the volume of 0.1 N KMnO_4_ used for titrating the blank, cm^3^; *V*_1_ is the average volume of KMnO_4_ used for titrating the working sample, cm^3^; *M*_e_ is the chemical equivalent of H_2_S_2_O_8_, (97 g eq mol^−1^); *V*_m_ is the total volume of synthesised analyte, cm^3^; *N* is the normality of KMnO_4_, mol-eq L^−1^; *V*_*t*_ is the volume of analyte aliquot taken for analysis, cm^3^.

Calculation of current efficiency, energy consumption, and sulfuric acid conversion for H_2_S_2_O_8_ production. The current efficiency (yield) (*η*_*t*_) for the obtained peroxodisulfuric acid was calculated using eqn (9):9
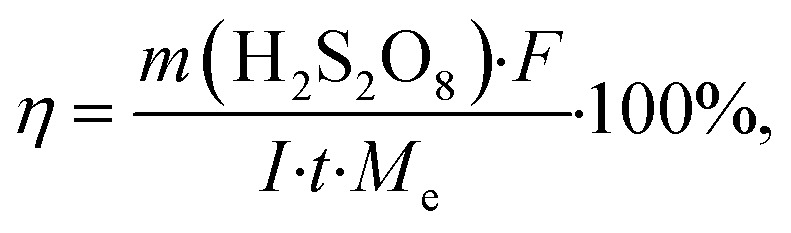
where, *η* is the average current efficiency in time *t*, %; *F* is the Faraday constant, 96485.3 C mol^−1^; *I* is current, A; *t* is electrolysis time, s.

The energy consumption per unit mass of H_2_S_2_O_8_ (*w*, Wh g^−1^) was determined using eqn (10):10
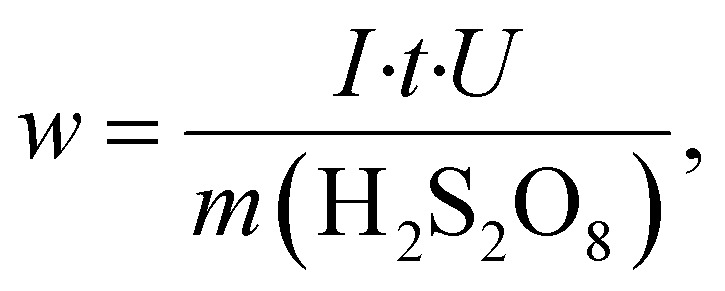
where *U* is the cell voltage, *V*.

The degree of sulfuric acid conversion to peroxodisulfuric acid (*X*, %) was calculated using eqn (11):11
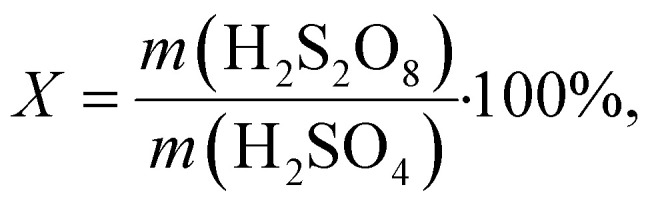
where *m*(H_2_SO_4_) is the mass of sulfuric acid taken for the electrolysis, g.

#### 2.3.1 Leaching methodology

Black mass (1.00 g) was treated with 10 cm^3^ of one of the selected leaching solutions, maintaining a solid-to-liquid ratio of 1 : 10. The solutions used included: aqua regia (HNO_3_ : HCl, 1 : 3), 2 M H_2_SO_4_ + 5 vol% H_2_O_2_, and synthesized and diluted 2 M H_2_S_2_O_8_. Aqua regia was freshly prepared before use by mixing concentrated HCl and HNO_3_ in a 3 : 1 volume ratio, and 2 M H_2_SO_4_ with 5% H_2_O_2_ was prepared by mixing 1.25 mL of concentrated H_2_SO_4_ (*ρ* = 1.8 g cm^−3^) with 0.5 cm^3^ of 37% H_2_O_2_.

Initial leaching was performed in an ultrasonic bath at 70 °C for 1 hour. After sonication, 25 cm^3^ of distilled water was added, and the suspension was stirred on a magnetic stirrer for an additional 12 h. The solid residue was separated by vacuum filtration, washed with distilled water, and the filtrate was diluted to a total volume of 250 cm^3^. Metal concentrations in the resulting solutions were determined using atomic absorption spectroscopy (AAS).

#### 2.3.2 Electrochemical cell

**Fig. 1:**
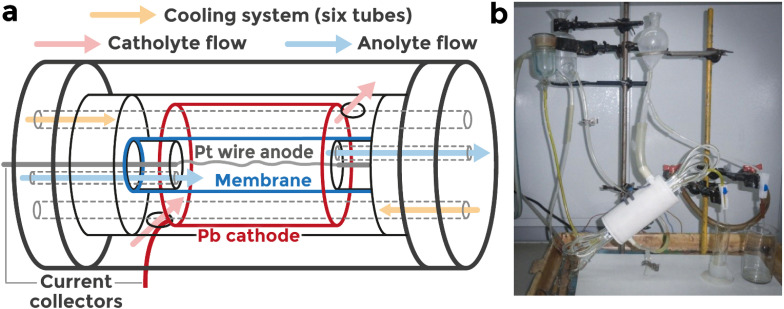
(a) Schematic and (b) photograph of the assembled flow electrolyser used in the study.

The flow-electrolyser used for peroxodisulfuric acid generation was made of Teflon and had a cylindrical shape. Inside the Teflon cylinder, the components were arranged coaxially (starting from the centre) as follows: a platinum anode, a membrane, and a lead cathode (Fig. 1a). The membrane was tightly fixed on Teflon spacers, which were inserted into the Teflon cylinder. This configuration separated the cathode and anode spaces inside the cell. The cathode compartment contained a cooling part consisting of six glass tubes. The working volume of the anode compartment was 18 cm^3^, and the cathode compartment 60 cm^3^. The anode current collector (Pb) was routed through the anolyte inlet port, while the cathode current collector (Pt) was routed through the catholyte inlet port (Fig. 1a).

The catholyte and anolyte were supplied by gravity feed (from reservoirs located above the electrolyser level) through the lower part of the electrolyser (the electrolyser was placed 45° based on the ground level, Fig. 1b), while the products of electrolysis were discharged from the top. The outlet ports were equipped with roller clamps for flow rate control (Fig. 1b).

The electrolyser was powered using an MPS-3005D DC power supply, and an additional voltammeter was installed for detailed monitoring of current and voltage during operation.

#### 2.3.3 Electrolysis methodology

To perform the electrolysis for peroxodisulfuric acid generation, the electrolyser was filled with catholyte and anolyte from thermostated reservoirs positioned above the cell and connected to it. The catholyte consisted of 4 M H_2_SO_4_, while the anolyte contained 4 M H_2_SO_4_ with the addition of ammonium thiocyanate (NH_4_CNS, 1 g dm^−3^). The electrolysis was carried out at a constant temperature of 10 °C. The flow rate was regulated using roller clamps. During operation, the liquid level in the reservoirs containing the initial working solutions (catholyte and anolyte) was maintained at a constant level to ensure stable hydrostatic pressure, thereby guaranteeing a consistent electrolyte flow rate. The experiment lasted from 2 to 3 hours, and every 30 minutes, a 1 cm^3^ sample of the anolyte product was collected to determine the concentration of the produced peroxodisulfuric acid by titration.

## 3 Results and discussion

### 3.1 Effect of electrolysis parameters on H_2_S_2_O_8_ generation

Based on our previous work on stationary electrolysis, the optimal current density range for generating peroxodisulfuric acid lies between 0.5 and 1.0 A cm^−2^. Under stationary conditions, exceeding this range results in rapid product accumulation in the bulk electrolyte, which increases parasitic decomposition reactions and leads to a sharp decline in current efficiency. In a flow-type electrolyser, these limitations are significantly mitigated. The continuous circulation of electrolyte through the anodic compartment reduces the residence time of the product in contact with the electrode surface, allowing the optimal current density window to be expanded to 0.5–1.5 A cm^−2^, or potentially higher. This broader range permits more flexible operational control, especially when targeting higher production rates without proportionally increasing energy losses. The electrolyte flow rate is another critical operational variable, determined by both the volume of the anodic compartment and the applied current density. For the current electrolyser design, the anodic compartment volume is 18 cm^3^, and the optimal flow rate corresponds to 1–3 anodic volumes per hour. This corresponds to a rate of 0.3–0.9 cm^3^ min^−1^, ensuring sufficient mass transfer while preventing excessive dilution of the product.

By carefully adjusting the current density and flow rate within these optimal ranges, it becomes possible to achieve the desired peroxodisulfuric acid concentration with minimal specific energy consumption. This operational flexibility is particularly advantageous when integrating the system into industrial-scale processes, where both efficiency and cost control are critical performance indicators.

**Fig. 2:**
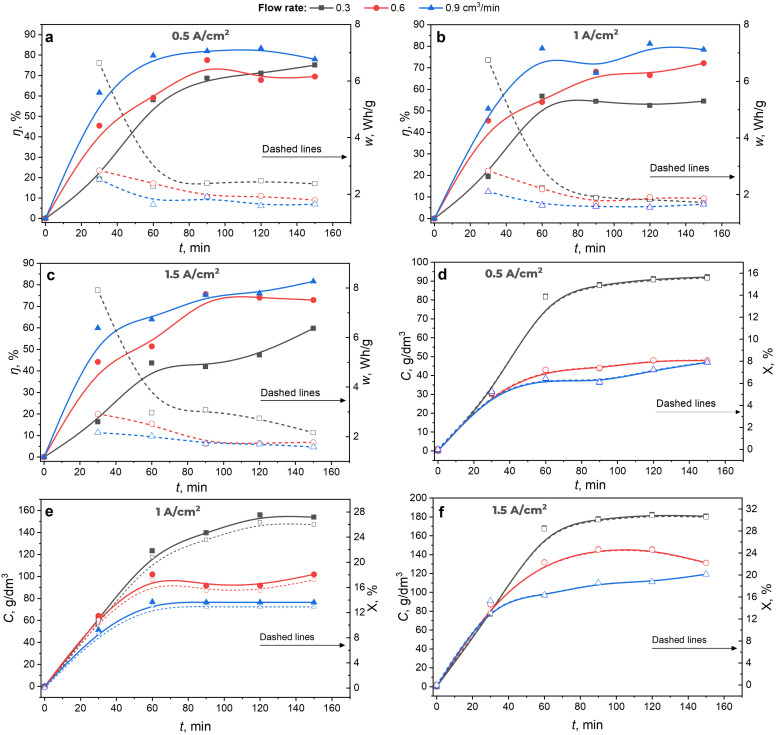
Dependence of current efficiency and specific energy consumption on time at different flow rates: (a) 0.5 A cm^−2^, (b) 1 A cm^−2^, (c) 1.5 A cm^2^; dependence of H_2_S_2_O_8_ concentration and H_2_SO_4_ conversion rate at different flow rates on time: (d) 0.5 A cm^−2^, (e) 1 A cm^−2^, (f) 1.5 A cm^−2^.

Fig. 2 presents the influence of current density and electrolyte flow rate on the main process parameters for peroxodisulfuric acid synthesis, including current efficiency (*η*, %), product concentration (C, g cm^−3^), and specific energy consumption (*w*, Wh g^−1^).

#### 3.1.1 Current efficiency (*η*)

For all current densities studied (0.5, 1.0, and 1.5 A cm^−2^), increasing the flow rate from 0.3 cm^3^ min^−1^ to 0.9 cm^3^ min^−1^ improved current efficiency (Fig. 2(a–c). At 0.5 A cm^−2^, *η* rose from 77.83% to 81.12%. At 1.0 A cm^−2^, the effect was more pronounced, with an increase from 52.77% to 76.05% increasing the flow rate from 0.3 cm^3^ min^−1^ to 0.9 cm^3^ min^−1^. A similar trend was observed at 1.5 A cm^−2^ (from 53.34% to 75.42%). This improvement is associated with enhanced heat removal resulting from ohmic losses (higher synthesis temperature – higher level of parasitic reactions) and faster mass transfer, which suppresses side reactions such as H_2_S_2_O_8_ hydrolysis and oxygen evolution.

#### 3.1.2 Conversion of H_2_SO_4_ to H_2_S_2_O_8_ (*X*)

At a constant flow rate, increasing current density logically increased the degree of conversion. At the lowest flow rate (0.3 cm^3^ min^−1^), *X* rose from 15.57% at 0.5 A cm^−2^ to 30.54% at 1.5 A cm^−2^ at 150 min of experiment. At the highest flow rate (0.9 cm^3^ min^−1^), the conversion values were lower, ranging from 7.92% to 20.13% due to the shorter residence time in the anodic compartment at 150 min of experiment. This illustrates a trade-off between rapid electrolyte renewal and sufficient retention time for product formation, enabling the prediction of the amount of remaining H_2_SO_4_.

#### 3.1.3 H_2_S_2_O_8_ concentration (*C*)

The highest concentrations were obtained at low flow rates. At 1.5 A cm^−2^ and 0.3 cm^3^ min^−1^, *C* reached 182.31 g dm^−3^, while at 0.9 cm^3^ min^−1^ it dropped to 119.16 g dm^−3^. A similar pattern occurred at 1.0 A cm^−2^ (155.83 g dm^−3^*vs.* 76.39 g dm^−3^). Thus, although high flow rates improve efficiency, they dilute the produced H_2_S_2_O_8_ concentration, which is also a key target parameter for leaching (with subsequent dilution).

#### 3.1.4 Specific energy consumption (*w*)

For all current densities, *w* decreased with increasing flow rate. At 1.0 A cm^−2^, energy consumption fell from 2.45 Wh g^−1^ at 0.3 cm^3^ min^−1^ to 1.70 Wh g^−1^ at 0.9 cm^3^ min^−1^, mainly due to lower ohmic resistance and the aforementioned suppression of parasitic reactions.

#### 3.1.5 Process optimisation

It should be emphasised that current efficiency in electrolysis is not a key factor, but rather a parameter directly linked to the specific energy consumption of the process. A decrease in current efficiency inevitably increases the energy required to produce a unit mass of product (H_2_S_2_O_8_). Therefore, optimising current efficiency as an independent target is not considered meaningful in this context. A more critical operational target is the generated H_2_S_2_O_8_ concentration, as this determines the main applicability of the solution, particularly the ability to dilute it to the required concentration for leaching operations. In this regard, the degree of conversion serves not as a primary optimisation parameter, but as a boundary condition defining the practical concentration limits. Concentrations of peroxodisulfuric acid significantly above or below this range may be impractical for process integration. Among all the evaluated metrics, the specific energy consumption, with its dependence on flow rate and current density, should be regarded as the key optimisation criterion. This parameter exhibits a strong correlation with electrolysis conditions. While increasing the flow rate can reduce energy consumption, excessive dilution of the product will eventually lower the degree of conversion to impractically low values. Under our experimental conditions, the optimal balance corresponds to a specific energy consumption of approximately 1.5 Wh g^−1^, peroxodisulfuric acid concentration of about 180 g L^−1^ (or 0.93 M), and a conversion degree near 30%.

The coaxial geometry of the presented electrolyser appears to enable relatively high current efficiencies at elevated current densities, which distinguishes it from most reported systems. Previous studies predominantly employed boron-doped diamond (BDD) anodes as an alternative to platinum. Michaud *et al.*^2^ and Balaji *et al.*^14^ achieved maximum current efficiencies of 75% at current densities of 200 mA cm^−2^ using BDD electrodes in undivided flow-type cells operating at 20–25 °C with 7–7.5 M H_2_SO_4_. Balaji *et al.* further investigated the effect of cathode material and anode-to-cathode area ratio, finding that a Ti cathode with an 8.75 : 1 area ratio yielded product concentrations up to 0.48 M.^14^ Davis *et al.* reported approximately 45% efficiency in a divided flow electrolyser operated at 300 mA cm^−2^.^15^ Their experiments using electrolyte recirculation in a closed system with a Nafion membrane separator achieved a maximum sulfate-to-persulfate conversion of 78% at an initial H_2_SO_4_ concentration of 0.77 M. The highest reported current efficiency of 95% was obtained by Serrano *et al.*,^3^ though at a relatively low current density of 23 mA cm^−2^. Kusama *et al.*^1^ achieved 89% efficiency using a WO_3_ anode at a total current of only 10 mA in a batch cell. Although conducted at low currents, this result suggests that cost-effective electrode materials, when paired with optimized cell design, may enable industrial-scale synthesis.

In comparison, our platinum-based system achieved 81% current efficiency at 0.5 A cm^−2^ and maintained 75% at 1.5 A cm^−2^. The ability to sustain reasonable efficiencies at current densities exceeding 1 A cm^−2^ suggests that the coaxial configuration may offer practical advantages for industrial-scale implementation, where high throughput and moderate energy consumption are both required. The product concentration achieved in our system is higher than concentrations reported in previous studies while operating at higher current densities than those reported in the literature.

### 3.2 Leaching black mass by synthesised H_2_S_2_O_8_

To evaluate the possible effectiveness of black mass (NMC + graphite, LLP « First Recycling ») leaching by synthesised H_2_S_2_O_8_, a comparative study was performed against two conventional leaching systems (aqua regia and H_2_SO_4_ + H_2_O_2_). For a fair comparison, the H_2_S_2_O_8_ solution was diluted twofold to match the H_2_O_2_ content and acid concentrations to those in the H_2_SO_4_ + H_2_O_2_ reference system, assuming that 1 M H_2_S_2_O_8_ yields 1 M H_2_O_2_ (hydrolysis) according to eqn (6) and (7).

**Fig. 3:**
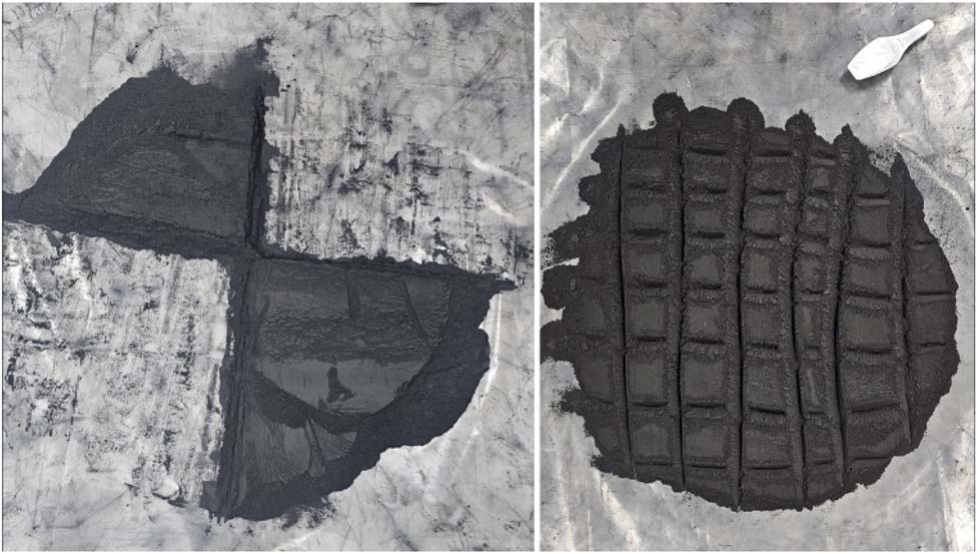
Photographs of the quartered black mass.

The starting black mass (total 16 kg) was homogenised using a standard quartering method to ensure representative sampling. The entire batch was first combined and thoroughly mixed at least 40 times to achieve maximum homogeneity. It was then divided into two equal portions, with mixing repeated after each division. This process continued until 1 kg fractions were obtained. The last fraction was subsequently quartered to obtain subsamples equivalent to 1/16 of the original batch for leaching tests (Fig. 3).

**Fig. 4:**
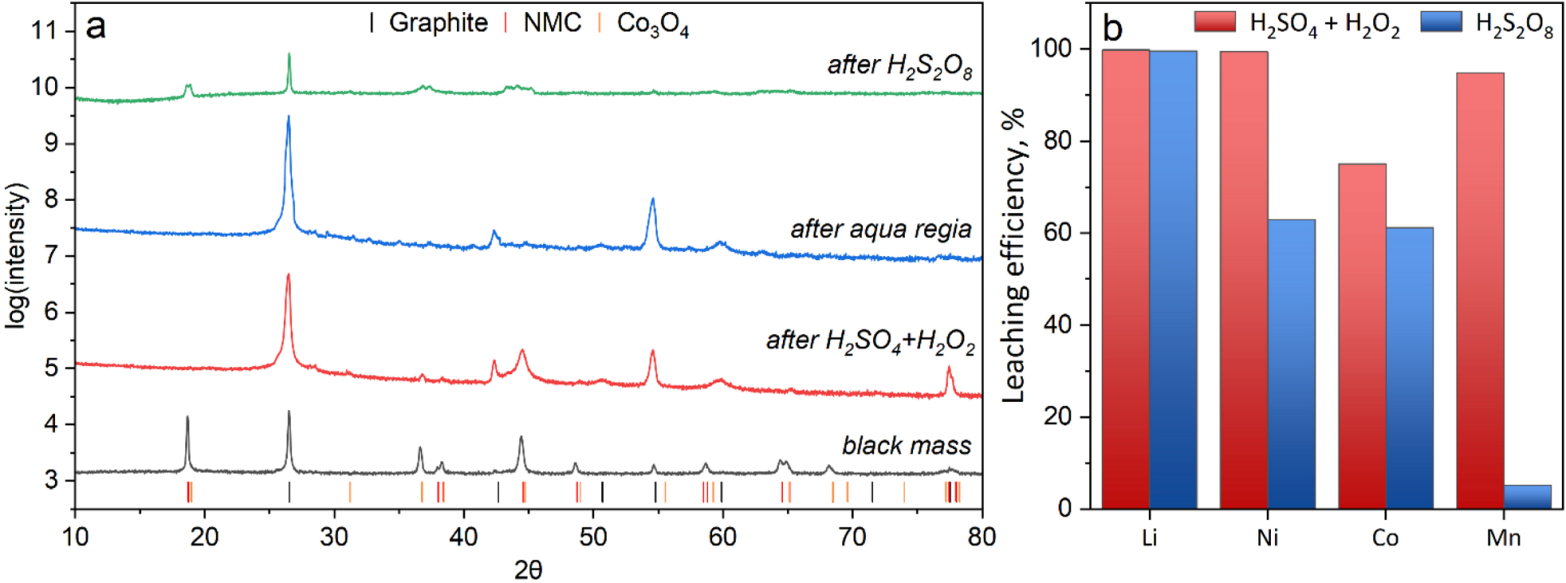
(a) XRD patterns of a representative probe of black mass and the remaining insoluble fraction after leaching in different etchants; (b) leaching efficiency in H_2_SO_4_ + H_2_O_2_ and H_2_S_2_O_8_ etching solutions relative to the aqua regia.

Leaching experiments with the abovementioned etchants were conducted according to the procedure described in Section 2.3. To evaluate the metal dissolution efficiency, AAS analysis of the filtrates obtained after etching was used, complemented by XRD of the insoluble residues (Fig. 4). XRD was employed with the intensity in the diffractograms (Fig. 4a) presented on a logarithmic scale to ensure reliable detection of low-intensity reflections. The analysis of the initial black mass confirmed its two-phase composition of graphite and NMC 811 cathode material (Li(Ni_0.8_Co_0.11_Mn_0.1_)O_2_ – NMC 811, PDF 12-0543), belonging to the *R*-3*m* space group.^16^ Leaching in aqua regia was used as a benchmark experiment demonstrating 100% dissolution of the cathode phase: its diffractogram shows only pure graphite remaining in the residue. The H_2_SO_4_ + H_2_O_2_ mixture showed a similar, yet slightly different result. After leaching in the standard H_2_SO_4_ + H_2_O_2_ mixture, the main reflections of the NMC phase disappear, indicating nearly complete dissolution; however, Rietveld refinement revealed the formation of a small amount of a secondary phase, cobalt oxide (Co_3_O_4_) (around 1–2%), in the residue, which points to the partial oxidative precipitation of dissolved cobalt. In contrast, after treatment with peroxodisulfuric acid (H_2_S_2_O_8_), the solid residue contains not only graphite but also a significant amount of undissolved NMC cathode material and the cobalt oxide Co_3_O_4_ phase. Thus, XRD analysis reveals fundamental differences in the mechanisms: the standard etchant provides almost complete dissolution of NMC, accompanied by the formation of small amount of cobalt oxide, whereas H_2_S_2_O_8_ acts as a less effective leaching agent, leading to partial dissolution of NMC and the parallel formation of Co_3_O_4_. No manganese-containing insoluble phases were identified, since the Mn content in the black mass (0.39 wt%) is below the detection limit of XRD analysis.

Table 1 presents the metal mass fraction determined by AAS analysis, which was performed on samples dissolved in various etchants, as well as the fraction of insoluble remaining material obtained through gravimetric analysis. With the assumption that all cathode materials were dissolved in aqua regia (only graphite present in the insoluble part, Fig. 4a), the specified cathode composition of NMC material in black mass is LiNi_0.89_Co_0.1_Mn_0.01_.

**Table 1:** Metals and insoluble fraction content in black mass determined by AAC analysis

	HNO_3_ + 3HCl	H_2_SO_4_ + H_2_O_2_	H_2_S_2_O_8_
*w*(Li), %	5.01	5.08	4,99
*w*(Ni), %	25.64	25.50	16.14
*w*(Co), %	3.95	2.97	2.42
*w*(Mn), %	0.39	0.37	0.02
Insoluble fraction	38.24	38.13	49.52

Based on the same assumption that the aqua regia solution dissolves the whole cathode part, the AAS analysis results demonstrate almost complete leaching (close to 100%) for the H_2_SO_4_ + H_2_O_2_ etchant, leaving nearly 25% of the Co content in insoluble state (Co_3_O_4_). In contrast, the H_2_S_2_O_8_-based etchant shows good Li extraction efficiency (99.6%), while Ni and cobalt do not exceed 61% with, poor Mn dissolution (5.1%) (Fig. 4b).

The poor dissolution of the cathode material in the proposed H_2_S_2_O_8_ etching solution necessitates consideration of the oxidation states and dissolution mechanisms specific to the NMC811 structure. In the discharged state, this cathode material contains predominantly nickel in the +3 oxidation state with a minor fraction in the +2 state, while cobalt remains in the +3 state and manganese in the +4 state.^17,18^ During cathode charge, nickel ions undergo oxidation from +2 to +3 state, followed by oxidation of both Ni^3+^ and Co^3+^ to their higher valence states, whereas Mn^4+^ shows no redox activity until complete delithiation.^18^ For successful metal recovery, the leaching process must dissolve these metals while converting their higher oxidation states to soluble forms.

**Fig. 5:**
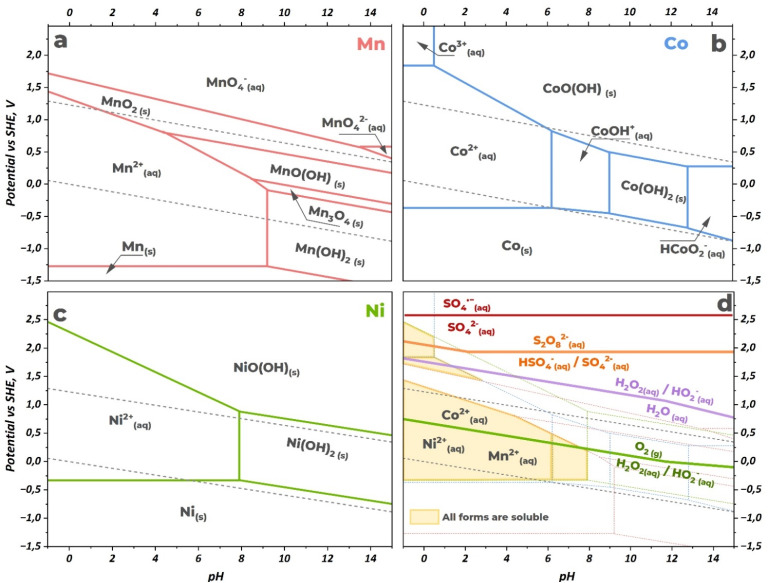
Pourbaix diagrams for (a) manganese, (b) cobalt, (c) nickel, and combined diagram with plotted redox lines for peroxodisulfuric acid and hydrogen peroxide as both oxidizing and reducing agents (d). The yellow shaded area indicates the region where all three elements exist in aqueous soluble forms. Diagrams were calculated according to thermodynamic equations presented in ref. 20. Calculations were performed at concentrations of 0.001 M for soluble forms of all metals and 1 M for peroxodisulfuric acid and hydrogen peroxide species. The sulfate radical position is shown as the minimum value of the reported range 2.6–3.1 V *vs.* SHE.^5^

The Pourbaix diagrams for the individual metals (Fig. 5a–c) reveal the specific conditions required for dissolution. Nickel exhibits broad solubility as Ni^2+^ across a wide range of acidic pH values. Cobalt dissolution can proceed through two pathways: reduction to Co^2+^ or stabilization of Co^3+^ under highly acidic and oxidizing conditions. Manganese can be dissolved either by substantial reduction to Mn^2+^ or by strong oxidation to soluble permanganate species (MnO_4_^−^, oxidation state +7).

Peroxodisulfuric acid possesses sufficient oxidizing power to thermodynamically drive the dissolution of all three metals. Moreover, during oxidation reactions or thermolysis, H_2_S_2_O_8_ can generate sulfate radicals 
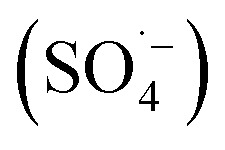
 as reactive intermediates, which exhibit even higher oxidizing potential. Fig. 5d shows that at low pH, the H_2_S_2_O_8_/HSO_4_^−^ couple lies within the stability regions of Co^3+^ and potential Mn^7+^ species while remaining compatible with Ni^2+^ dissolution. However, in the presence of such powerful oxidizing species, both nickel and cobalt would likely be stabilized in their highest +4 oxidation states as insoluble oxide phases, hindering their dissolution. The practical leaching efficiency falls short of the H_2_SO_4_ + H_2_O_2_ system, particularly for manganese. This limitation probably stems from kinetic and mechanistic factors. The oxidation of manganese to soluble permanganate species requires alkaline conditions and is industrially performed through a two-stage process: first oxidation to manganate (VI) by oxygen in a concentrated KOH solution, then electrochemical oxidation to permanganate.^19^

The effectiveness of H_2_S_2_O_8_ as an indirect source for the desired leaching agents (H_2_SO_4_ and H_2_O_2_) requires activation of the peroxide bond in the peroxydisulfate anion (S_2_O_8_^2−^). However, the formation of these end products is not instantaneous, and the hydrolysis process proceeds relatively slowly even at elevated temperatures. The partial leaching efficiency observed in Fig. 4b likely results from the gradual hydrolysis of H_2_S_2_O_8_ to H_2_SO_4_ and H_2_O_2_ according to reactions (6 and 7), rather than direct oxidative action of the parent acid.

The superior leaching performance of H_2_O_2_ is attributed to its dual redox nature. While capable of oxidation (*E*° = 1.76 V *vs.* SHE^20^), its role as a reducing agent (*E*° = 0.69 V *vs.* SHE^20^) proves crucial for black mass applications. Notably, Fig. 5d shows that under strongly acidic conditions, the oxidizing potential of H_2_O_2_ approaches that of H_2_S_2_O_8_, yet the significant difference in leaching efficiency between the two systems further illustrates the preference for reductive over oxidative leaching pathways. The redox potential of the H_2_O_2_/O_2_ couple positions it within the stability window of soluble divalent metal ions (Fig. 5d). This reductive pathway allows effective conversion of insoluble Co^3+^ and Mn^4+^ oxides to soluble Co^2+^ and Mn^2+^ species. This approach bypasses the kinetic limitations associated with high-temperature oxidative dissolution, explaining the superior performance of H_2_SO_4_ + H_2_O_2_ mixtures for mixed-oxidation-state cathode materials.

The high lithium extraction efficiency (99.6%) observed with H_2_S_2_O_8_ requires separate consideration due to the dual nature of lithium presence in black mass. Lithium compounds from residual electrolyte components readily dissolve in the acidic medium, converting to soluble ionic forms. Simultaneously, lithium incorporated within the cathode structure undergoes chemical deintercalation in the presence of peroxodisulfuric acid through oxidation of the host material, analogous to the electrochemical charging process but driven by chemical rather than electrical potential.^21,22^ This dual mechanism ensures efficient lithium extraction regardless of its initial chemical environment. The observed lithium recovery represents co-dissolution rather than selective extraction, as both processes occur independently of the specific leaching mechanism employed for the transition metals.

As a result, the proposed etching solution based on electrochemically synthesised H_2_S_2_O_8_ in the flow electrolyser, which can be installed on the spot at the factory, shows poor extraction efficiency for transition metals (around 60% for Ni and Co and 5% for Mn, especially for cathodic composition NMC 811) and high etching efficiency for Li (close to 100%). Some works^6,10,11^ claim that this is a case of selective Li extraction, but in our view, partial NMC dissolution with transition metal extraction in the solution cannot be considered selective, as it requires additional steps of separation.

## 4 Perspective remarks

In this work, we demonstrated an electrochemical synthesis of peroxodisulfuric acid and evaluated its potential for black mass leaching. While the results show inferior performance compared to the conventional H_2_SO_4_ + H_2_O_2_ system for transition metal extraction, complete lithium recovery (99.6%) was achieved. The high lithium extraction efficiency can be attributed to the strong oxidizing properties of H_2_S_2_O_8_, which enables chemical deintercalation from the cathode structure analogous to electrochemical charging.^21,22^

The limited transition metal extraction appears to result from incomplete hydrolysis of H_2_S_2_O_8_ under the experimental conditions employed. While the ultimate hydrolysis products of H_2_S_2_O_8_ are indeed H_2_SO_4_ and H_2_O_2_ (eqn (6) and (7)), the presence of unreacted peroxodisulfuric acid maintains a strongly oxidizing environment. This prevents H_2_O_2_ from functioning as the reducing agent necessary for dissolving higher oxidation state metals (Ni^3+^, Co^3+^, Mn^4+^) into their soluble divalent forms.

**Fig. 6:**
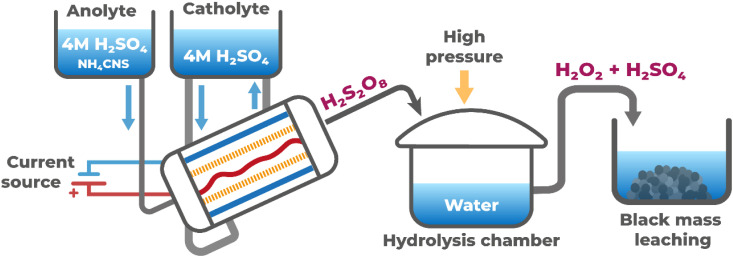
Proposed three-stage industrial process for black mass leaching using electrochemically generated peroxodisulfuric acid.

Despite these direct application limitations, a modified three-stage industrial process could potentially overcome the observed challenges (Fig. 6). The first stage would employ *in situ* electrochemical H_2_S_2_O_8_ generation using the flow electrolyser design described in this work. The second stage would focus on controlled hydrolysis to achieve complete conversion to H_2_SO_4_ + H_2_O_2_. The final stage would involve conventional reductive leaching of the black mass using the generated H_2_SO_4_/H_2_O_2_ mixture.

The hydrolysis step requires careful optimization. According to Le Chatelier's principle, water addition shifts the equilibrium toward complete hydrolysis products (eqn (6) and (7)). However, hydrogen peroxide is inherently unstable and decomposes according to:

12 2H_2_O_2_ → 2H_2_O + O_2_

To minimize H_2_O_2_ losses, the hydrolysis should be conducted under elevated pressure to suppress oxygen evolution and maintain dissolved oxygen levels. Moderate temperature elevation could accelerate hydrolysis kinetics, though this must be carefully balanced against the increased H_2_O_2_ decomposition rate at higher temperatures.^23^

This approach offers several practical advantages for industrial implementation. On-site H_2_S_2_O_8_ generation eliminates the safety hazards and logistical challenges associated with concentrated H_2_O_2_ transport and storage^24^ as in other H_2_O_2_ industrial synthesis methods.^25^ The electrochemical process also enables precise stoichiometric control of the final H_2_SO_4_ : H_2_O_2_ ratio by adjusting synthesis and hydrolysis parameters for specific black mass chemistries.

Another important consideration is the ability of peroxodisulfuric acid to destroy organic contaminants present in spent battery materials. These include carbonate-based electrolyte solvents and lithium salts with organic anions, which can interfere with subsequent hydrometallurgical processes. A pre-treatment step using dilute H_2_S_2_O_8_ solutions after mechanical size reduction could simultaneously accomplish organic pollutant destruction^5^ and partial lithium extraction, improving overall process efficiency.

## 5 Conclusions

In this work, we demonstrate the electrochemical synthesis of peroxodisulfuric acid in a coaxial flow-type electrolyser and evaluate its potential application for leaching spent lithium-ion battery black mass (NMC 811 type). By varying the main technological parameters (current density and flow rate), we optimised energy consumption and product concentration, balancing the conversion rate of H_2_SO_4_ for further utilisation in the leaching process.

Leaching experiments revealed a clear distinction between the performance of H_2_S_2_O_8_ and conventional agents. Aqua regia enabled the complete dissolution of the cathode fraction, whereas the H_2_SO_4_/H_2_O_2_ system provided nearly complete recovery of Li, Ni, Co, and Mn, with only minor precipitation of Co_3_O_4_. In contrast, direct H_2_S_2_O_8_ application resulted in almost quantitative lithium recovery (99.6%) but limited transition metal dissolution (approximately 61% for Ni and Co, and only 5% for Mn). Structural analysis indicated that the strongly oxidising environment of H_2_S_2_O_8_ stabilises insoluble high-valent oxides, which hinders reductive dissolution pathways essential for transition metal recovery.

Overall, electrochemically synthesised H_2_S_2_O_8_ shows promise as part of a hybrid recycling strategy, combining the benefits of on-site oxidant generation with established hydrometallurgical processes, and thereby contributing to safer, greener, and more efficient recycling of end-of-life lithium-ion batteries.

## Author contributions

Aigerim Tazhibayeva: conceptualization, methodology, investigation, funding acquisition, writing – original draft, writing – review & editing. Altynai Tanash: investigation, validation, formal analysis, visualization, writing – review & editing. Yaroslav Zhigalenok: methodology, investigation, formal analysis, data curation, visualization, writing – original draft. Saken Abdimomyn: investigation, resources, validation, formal analysis, writing – review & editing. Seiilbek Malik: investigation, data curation, formal analysis, visualization, writing – review & editing. Kaiyrgali Zhumadil: methodology, investigation, resources, validation, writing – review & editing. Sergey Nechipurenko: supervision, project administration, resources, writing – review & editing. Fyodor Malchik: conceptualization, methodology, supervision, data curation, visualization, writing – review & editing.

## Conflicts of interest

There are no conflicts to declare.

## Supplementary Material

RA-015-D5RA06474K-s001

## Data Availability

The experimental data supporting this article have been included as part of the supplementary information (SI). Supplementary information: processed datasets for the electrochemical synthesis parameters, including current efficiency, specific energy consumption, H_2_S_2_O_8_ concentration, and H_2_SO_4_ conversion rates; data for the leaching experiments, such as the leaching efficiencies and the raw XRD pattern data. The Pourbaix diagrams are theoretical calculations based on the methodology detailed in the figure caption. Further calculation details related to these graphs are available from the corresponding author upon reasonable request. See DOI: https://doi.org/10.1039/d5ra06474k.

## References

[cit1] Kusama H., Kodera M., Sayama K. (2023). ChemistrySelect.

[cit2] Michaud P. A., Mahé E., Haenni W., Perret A., Comnineiiis C. (2000). Electrochem. Solid-State Lett..

[cit3] Serrano K., Michaud P. A., Comninellis C., Savall A. (2002). Electrochim. Acta.

[cit4] Ike I. A., Linden K. G., Orbell J. D., Duke M. (2018). Chem. Eng. J..

[cit5] Lee J., Von Gunten U., Kim J. H. (2020). Environ. Sci. Technol..

[cit6] Lv W., Wang Z., Zheng X., Cao H., He M., Zhang Y., Yu H., Sun Z. (2020). ACS Sustain. Chem. Eng..

[cit7] Lin D., Fu Y., Li X., Wang L., Hou M., Hu D., Li Q., Zhang Z., Xu C., Qiu S., Wang Z., Boczkaj G. (2022). J. Hazard. Mater..

[cit8] Mayyas A., Steward D., Mann M. (2019). Sustainable Mater. Technol..

[cit9] Melchor-Martínez E. M., Macias-Garbett R., Malacara-Becerra A., Iqbal H. M. N., Sosa-Hernández J. E., Parra-Saldívar R. (2021). Case Stud. Chem. Environ. Eng..

[cit10] Ji Z. Y., Zhao M. Y., Zhao Y. Y., Liu J., Peng J. L., Yuan J. S. (2017). Solid State Ionics.

[cit11] Yuan J. S., Yin H. B., Ji Z. Y., Deng H. N. (2014). Ind. Eng. Chem. Res..

[cit12] Meshram P., Abhilash, Pandey B. D., Mankhand T. R., Deveci H. (2016). Jom.

[cit13] Moon S., Chae W., Jun B. M., Yoon Y., Rho H. (2025). Sep. Purif. Technol..

[cit14] Balaji S., Muthuraman G., Moon I. S. (2015). J. Hazard. Mater..

[cit15] Davis J. R., Baygents J. C., Farrell J. (2014). J. Appl. Electrochem..

[cit16] Jeong M., Kim H., Lee W., Ahn S. J., Lee E., Yoon W. S. (2020). J. Power Sources.

[cit17] Hsieh I.-T., Wu Y., Li B., Qi Y. (2024). Solid State Ionics.

[cit18] White J. L., Gittleson F. S., Homer M., El Gabaly F. (2020). J. Phys. Chem. C.

[cit19] Singh N., Lee D. G. (2001). Org. Process Res. Dev..

[cit20] Schweitzer G. K. and Pesterfield L. L., The Aqueous Chemistry of the Elements, Oxford University Press, 2010

[cit21] Kurbatov A. P., Malchik F. I., Galeyeva A. K., Davydchenko D. S., Rakhimova A. K., Lepikhin M. S., Kamysbayev D. K. (2018). Russ. J. Electrochem..

[cit22] Malchik F., Kurbatov A., Galeyeva A., Kamysbaev D., Marincas A. H. (2017). Appl. Sci..

[cit23] Lin C. C., Smith F. R., Ichikawa N., Baba T., Itow M. (1991). Int. J. Chem. Kinet..

[cit24] Siahrostami S. (2023). Chem Catal..

[cit25] Shin H., Lee S., Sung Y. E. (2023). Curr. Opin. Electrochem..

